# Supplementary material for: Chd4 remodels chromatin to control retinal cell type specification and lineage termination
Source: Development. 2025 Sep 18;152(20):dev204697. doi: 10.1242/dev.204697 (PMC12517350; doi:10.1242/dev.204697)
Supplement: Supplementary information [file develop-152-204697-s1.pdf]

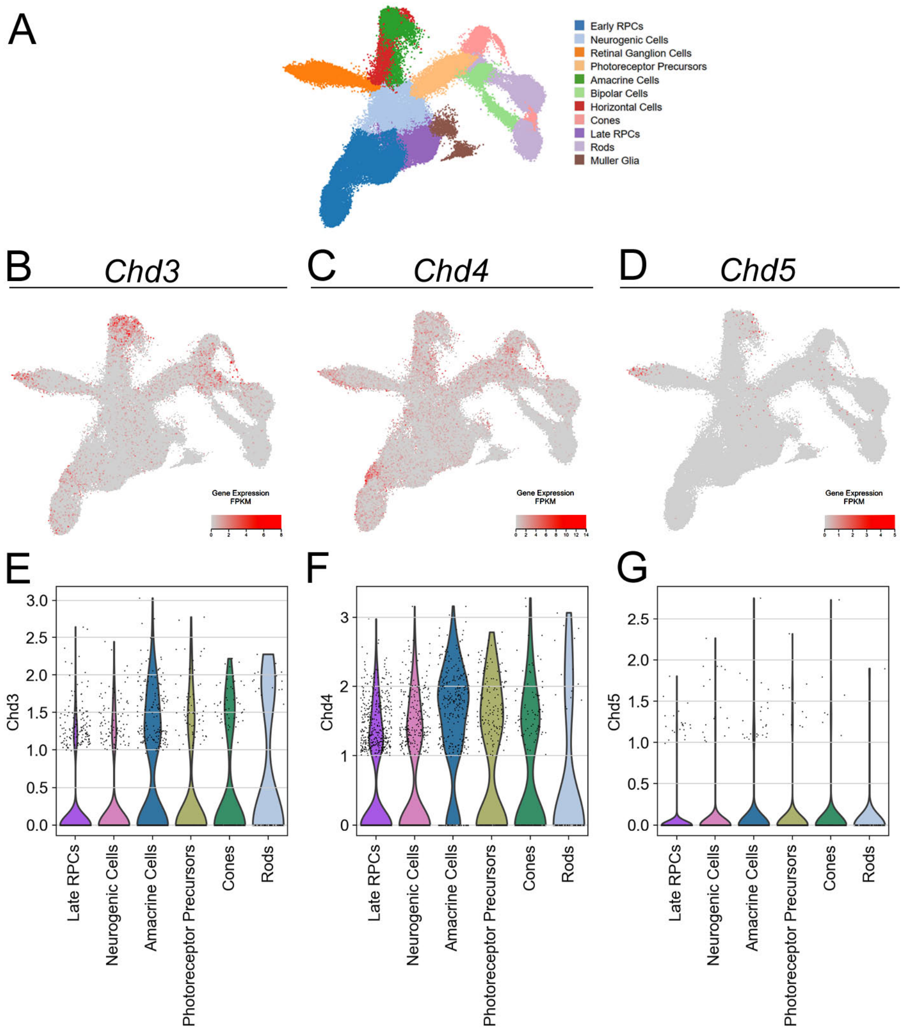

Fig. S1. Expression dynamics of *Chd3/4/5* during retinal development. (A) UMAP projection of retinal developmental trajectories from a previously published scRNA-seq dataset (Clark et al., 2019). (B-D) The expression of individual *Chd* paralogs during retinal development from the same atlas as indicated. (E-G) Violin plots displaying *Chd3* (E), *Chd4* (F), and *Chd5* (G) expression in retinal cell types as determined by scRNA-seq data from P1 control samples.

Development • Supplementary information

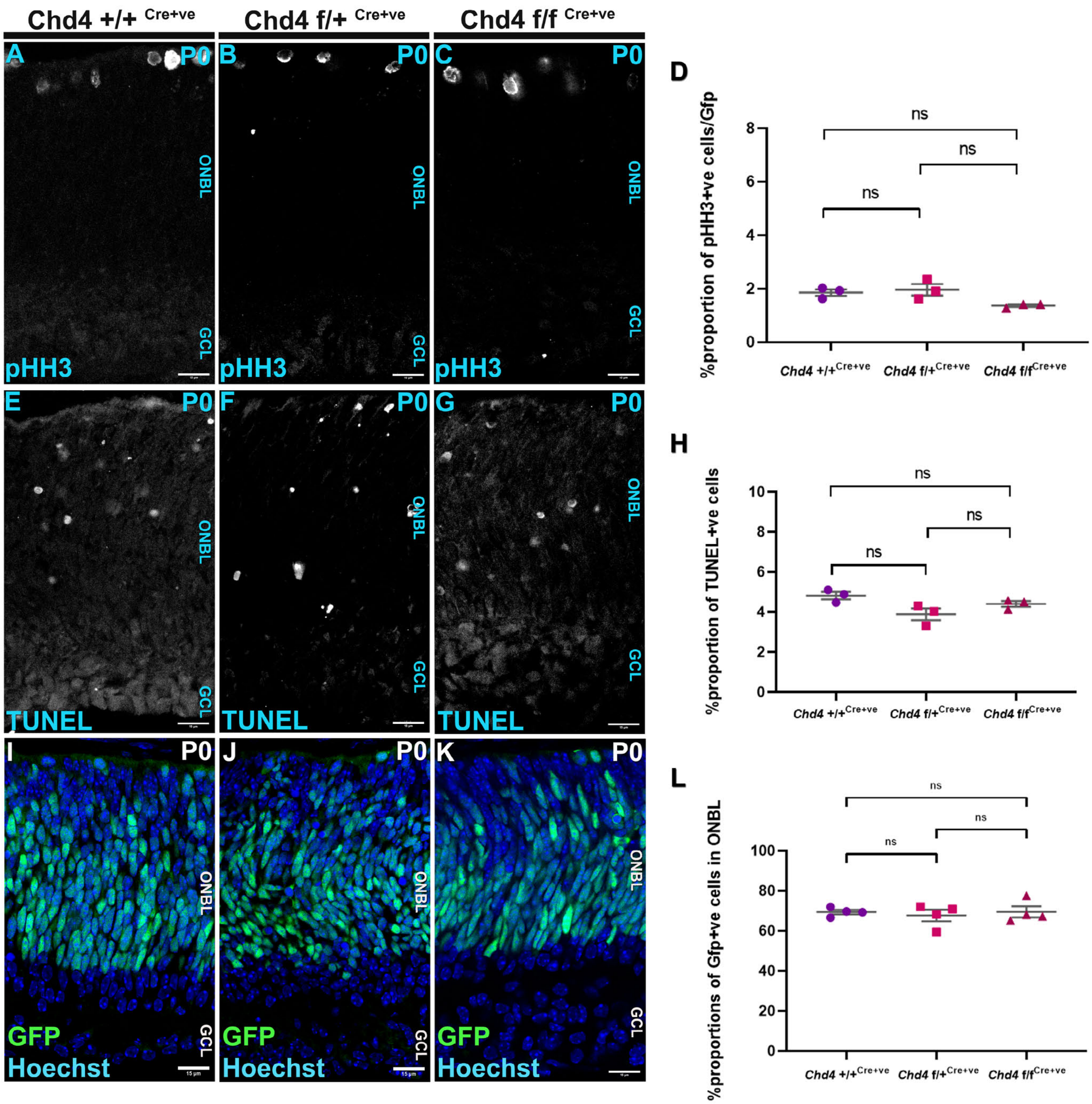

Fig. S2. *Chd4* cKO does not affect proliferation or viability at P0. (A-C) Wt, chet, and cKO retinal sections were stained with the mitotic marker phospho-histone H3. (D) Percentage of pHH3+ cells at P0. (E-H) TUNEL assay to quantify apoptotic cells. (I-K) GFP staining to mark Chx10+ progenitors. (L) Using GFP as a proxy for RPCs, the progenitor pool as quantified between the different genotypes. All data are presented as mean ±SEM. ns: not significant by one-way ANOVA with Tukey's multiple comparison test. ONBL: outer neuroblastic layer; GCL: ganglion cell layer. Scale bar = 10 microns.

Development • Supplementary information

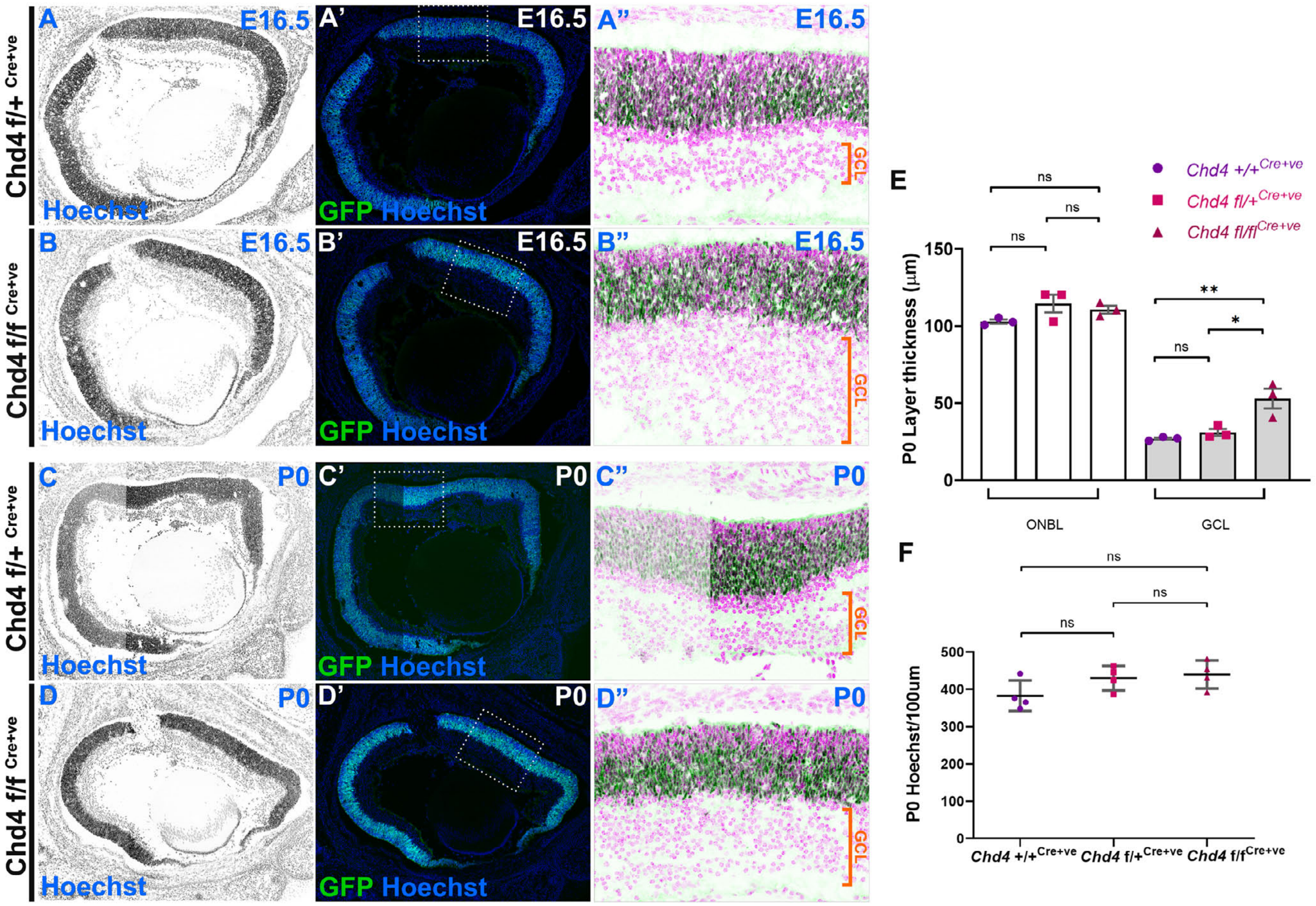

Fig. S3. *Chd4* cKO leads to embryonic expansion of the ganglion cell layer and lamination defects. (A-B″) E16.5 chet and cKO whole eye sections were stained with Hoechst and visualized for GFP epifluorescence . (C-D″) P0 chet and cKO whole eye sections were stained with Hoechst and visualized for GFP epifluorescence. (E) Quantification of individual layer thickness between the different genotypes. (F) Total Hoechst was quantified and compared across the three genotypes. All data are presented as mean ±SEM. p-value *p<0.05, **p<0.005, ns: not significant by one-way ANOVA with Tukey's multiple comparisons test.

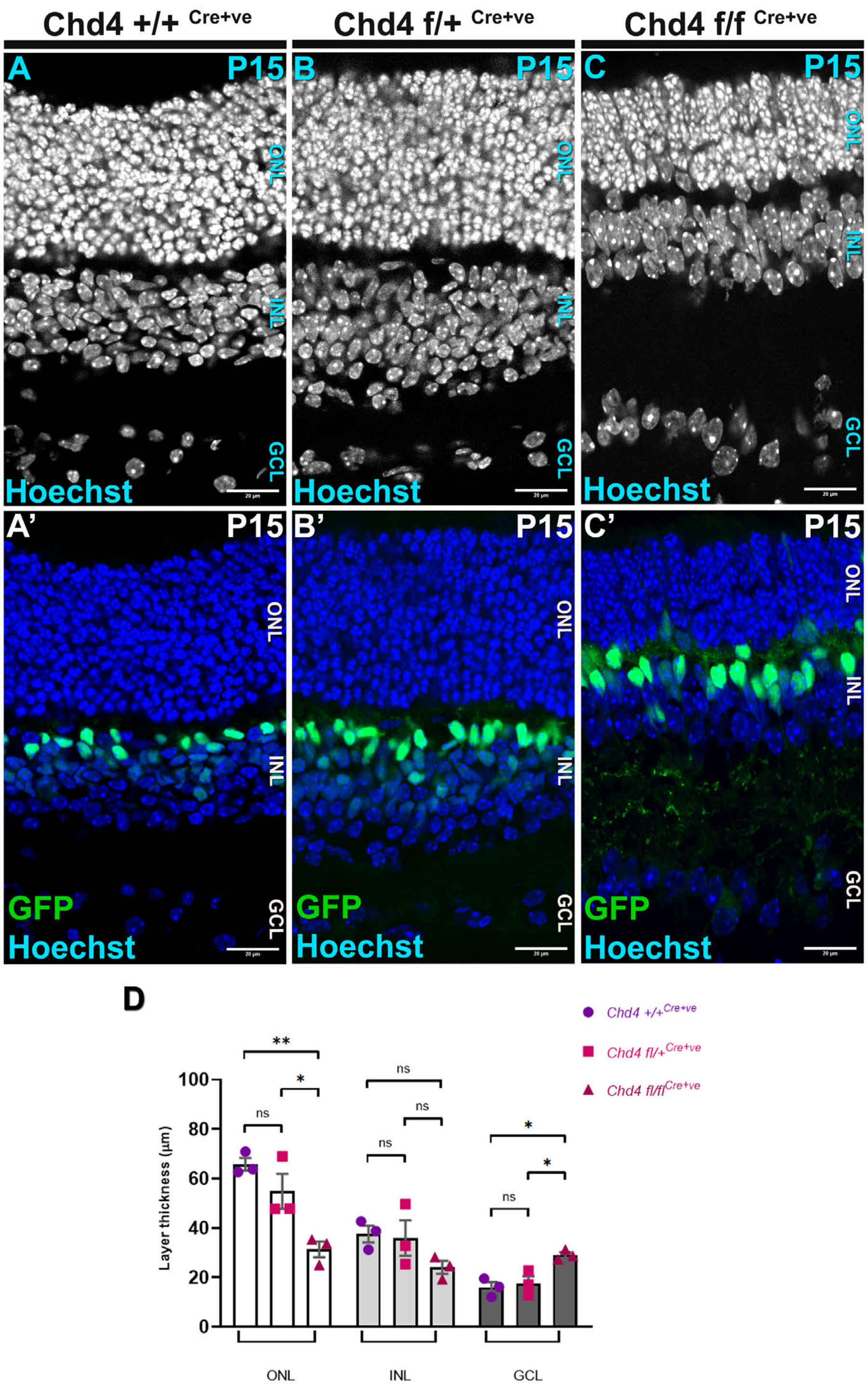

Fig. S4. Effect of *Chd4* cKO on P15 retinal histology. (A-C') Wt, chet and cKO retinal sections were stained with Hoechst to mark the cell bodies in the different retinal layers. (D) Quantification of individual layer thickness between the different genotypes. All data are presented as mean ±SEM. p-value *p<0.05, **p<0.005, ns=not significant by one-way ANOVA with Tukey's multiple comparisons test. ONL: outer nuclear layer; INL: inner nuclear layer; GCL: ganglion cell layer. Scale bar = 10 microns.

Development • Supplementary information

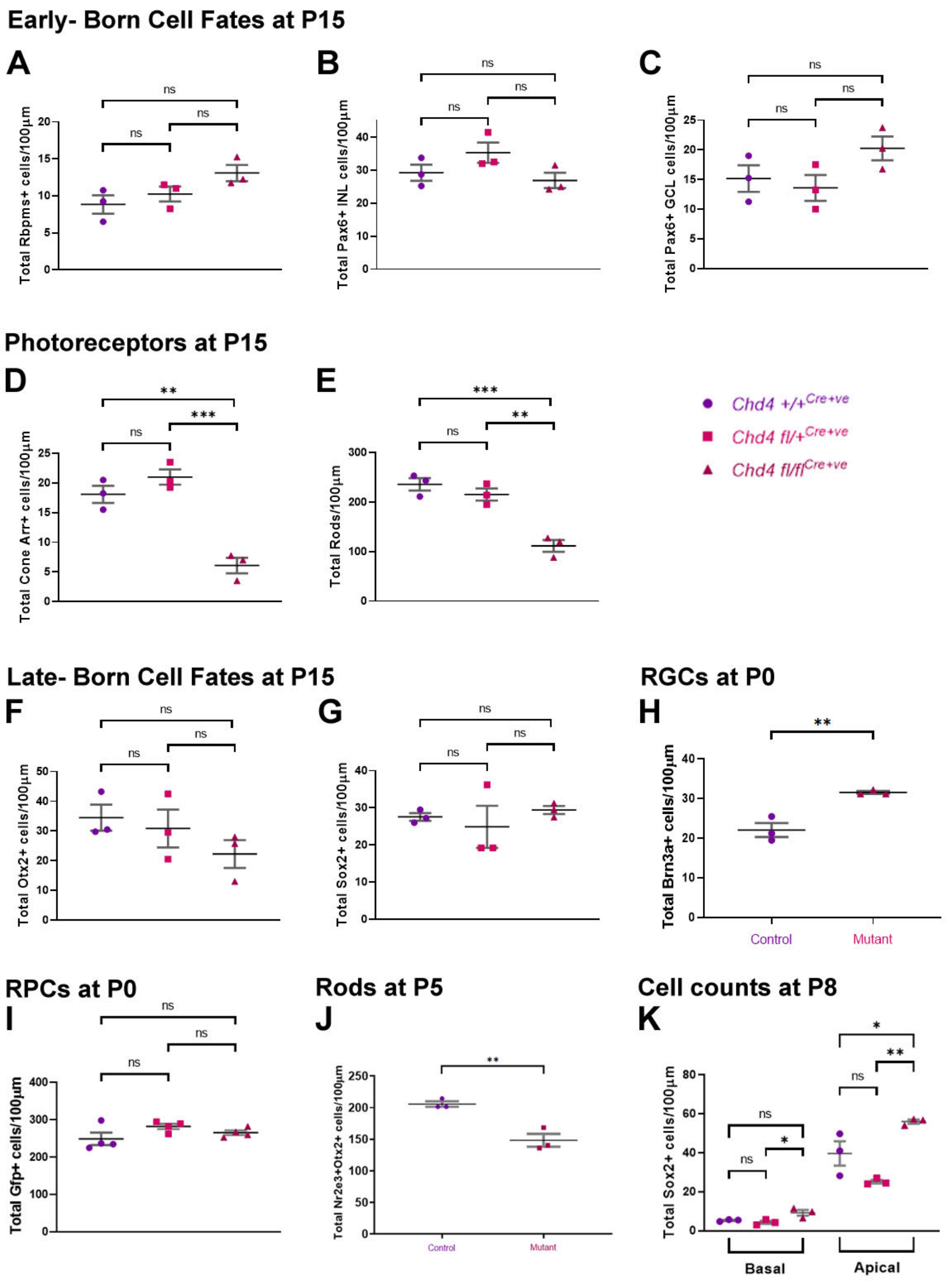

Fig. S5. Absolute cell numbers counted in hundred micron-wide bins. (A-G) Data from Fig. 3 re-expressed in absolute cell counts. (A) Rbpms+ RGCs. (B, C) Pax6+ amacrines (B; INL) along with RGCs (C; GCL). (D) Cone arrestin+ cones. (E) GFP-negative/cone-arrestin negative ONL cells were counted as rods. (F) Brightly positive Otx2 cells were counted as bipolars. (G) Radially polarized Sox2+ cells were counted as Müller glia. (H, I) Data from Fig. 4 re-expressed in absolute counts. (H) Brn3a+ RGCs. (I) GFP+ RPCs. (J, K) Data from Fig. 5 re-expressed as absolute counts. (J) Nr2e3+/Otx2+ rods. (K) Sox2+ cells. All data are presented as mean ±SEM. *p<0.05, **p<0.005, ***p<0.0005, ns=not significant by one-way ANOVA with Tukey's multiple comparison test.

Development • Supplementary information

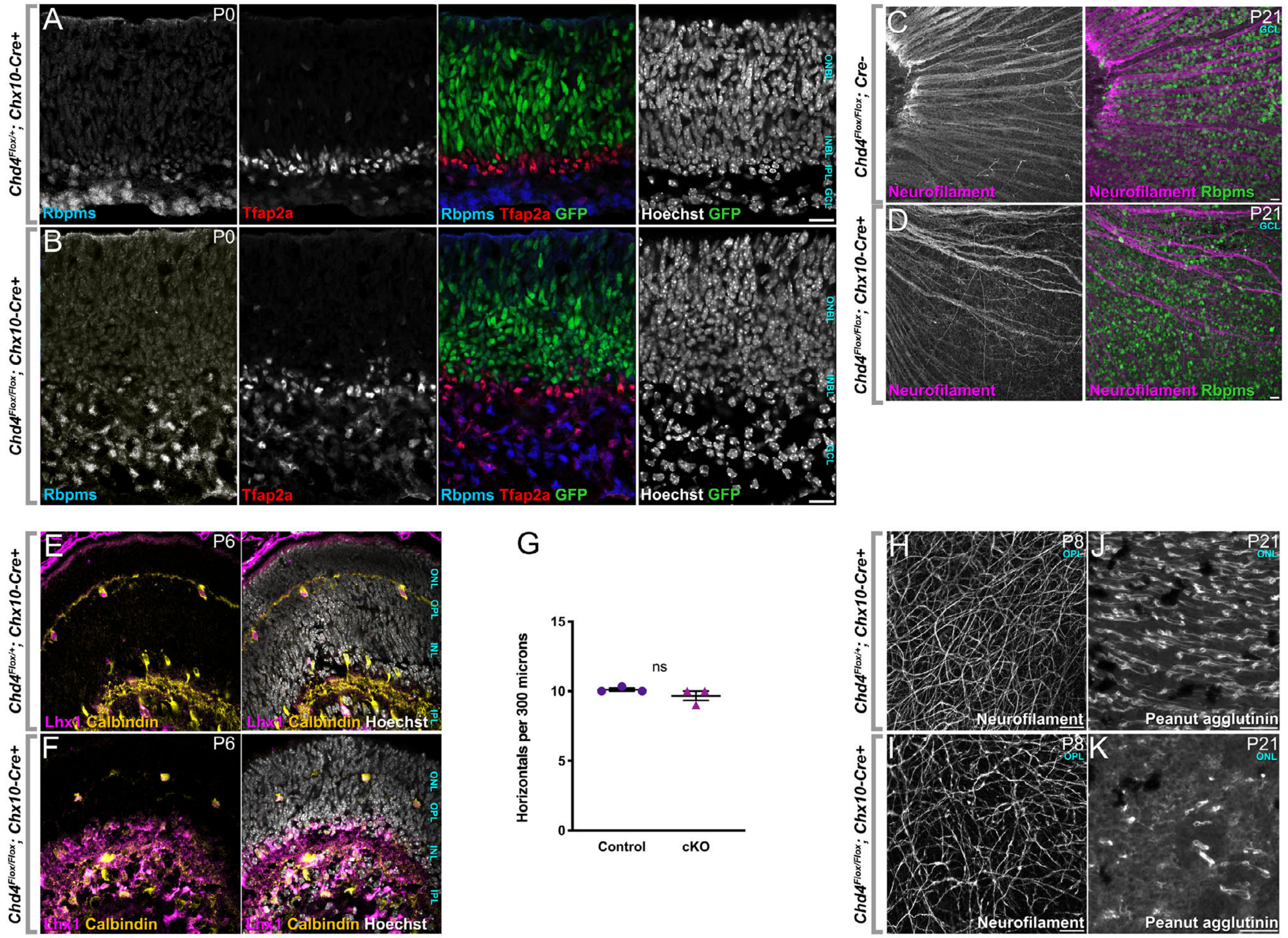

Fig. S6. Early-born cell type markers in the *Chd4* cKO. (A, B) P0 chet (A) and cKO (B) retinas co-stained with the ganglion cell marker Rbpms and the amacrine marker Tfap2a. Scale bar = 20 microns. (C, D) P21 wild-type (C) or cKO (D) wholemount stainings for the RGC markers Rbpms and neurofilament. At the level of the GCL and nerve fiber layer, neurofilament specifically marks RGC axons. (E, F) P6 chet (C) and cKO (D) retinas co-stained with the horizontal marker Lhx1 and the horizontal/amacrine marker calbindin. (G) Quantitation of Lhx1+ horizontal cells counted in 300 micron bins at P5. (H, I) P8 chet (E) and cKO (F) retinas stained in wholemount for neurofilament. Images depict single z-planes captured at the level of the outer plexiform layer, where neurofilament specifically marks horizontal cells. Scale bar = 30 microns. (J, K) P21 chet (J) and cKO (K) retinas stained in wholemount for peanut agglutinin. Images consist of extended focus composites at the level of the outer nuclear layer, where peanut agglutinin marks cone outer segments. Scale bar = 30 microns. ONBL: outer neuroblastic layer; ONL: outer nuclear layer; INL: inner nuclear layer; GCL: ganglion cell layer; OPL: outer plexiform layer; IPL: inner plexiform layer.

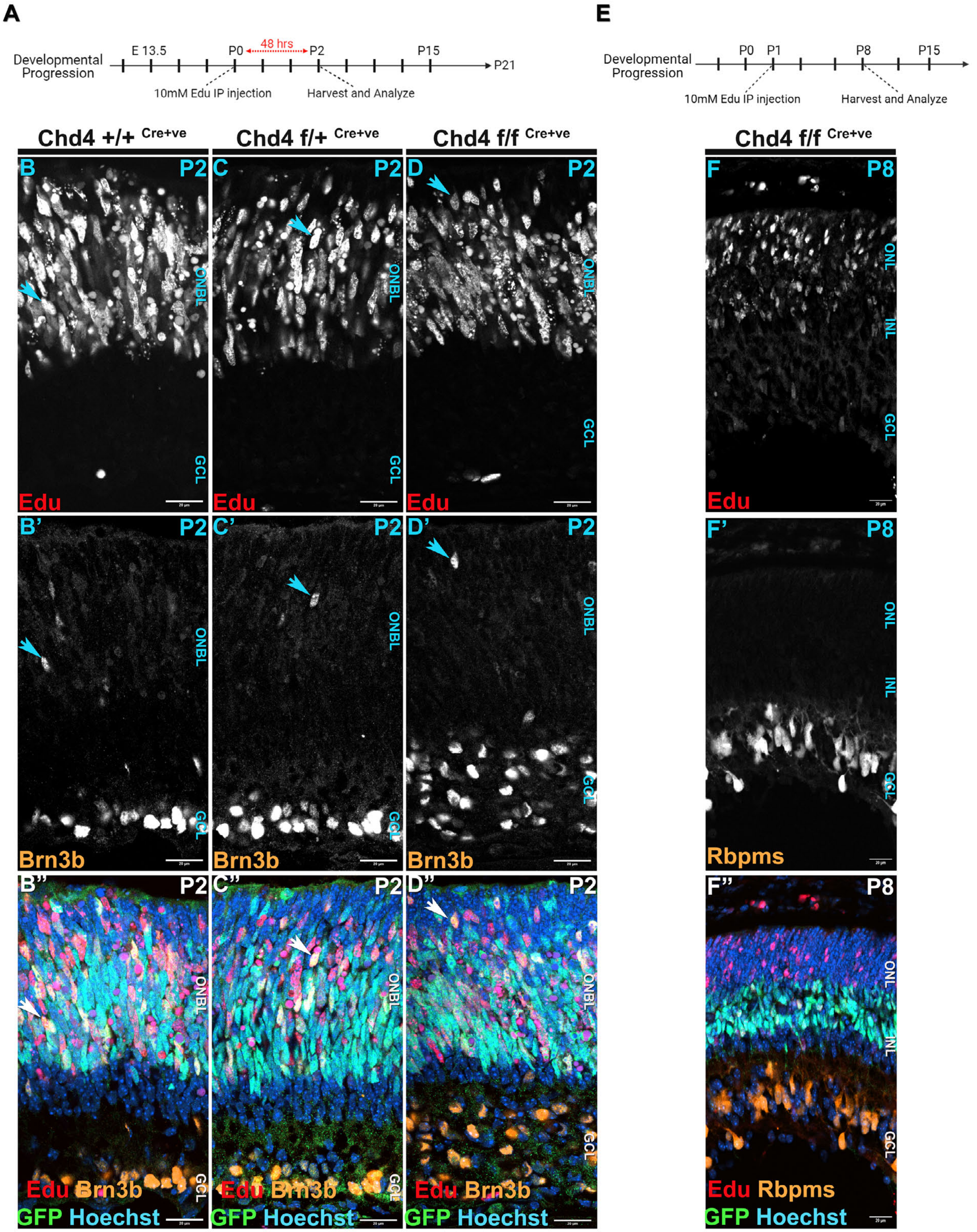

Fig. S7. RGCs are overproduced within their normal birth window in *Chd4* cKO retinas. (A) Schematic of 48-hours Edu birthdating assay. (B-D″) Wt, chet, and cKO retinas were stained with EdU and and Brn3b. Arrows indicate cells that are double positive for EdU and Brn3b. (E) Schematic of 7-day Edu birthdating assay. (F-F″) cKO retina was stained with EdU and and Rbpms. Scale bar = 10 microns.

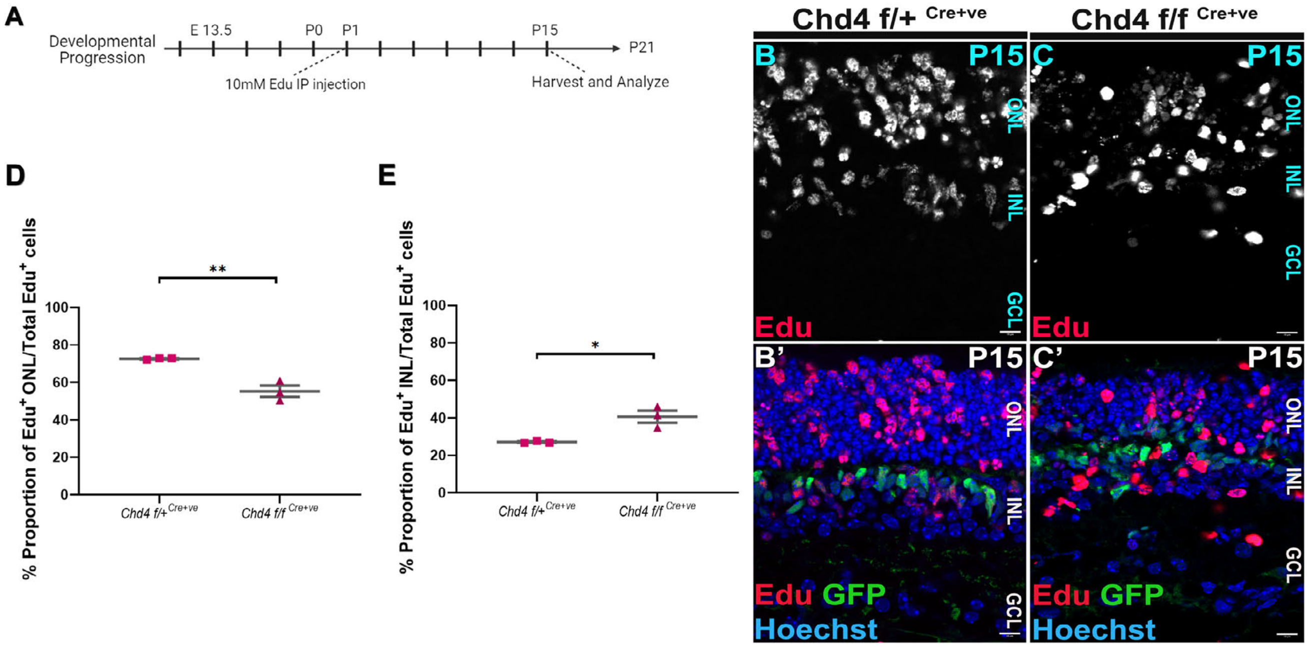

Fig. S8. Chd4 promotes the development of rod photoreceptors. (A) EdU was injected at P1 and retinas were harvested at P15. (B, C) EdU+ birthdated cells in cHet (B) versus cKO (C) retinas. (D, E) Quantification of the proportion EdU+ cells divided between the ONL (D) and INL (E). *p<0.05, **p<0.005, by Student's t test. Scale bar = 10 microns.

Development • Supplementary information

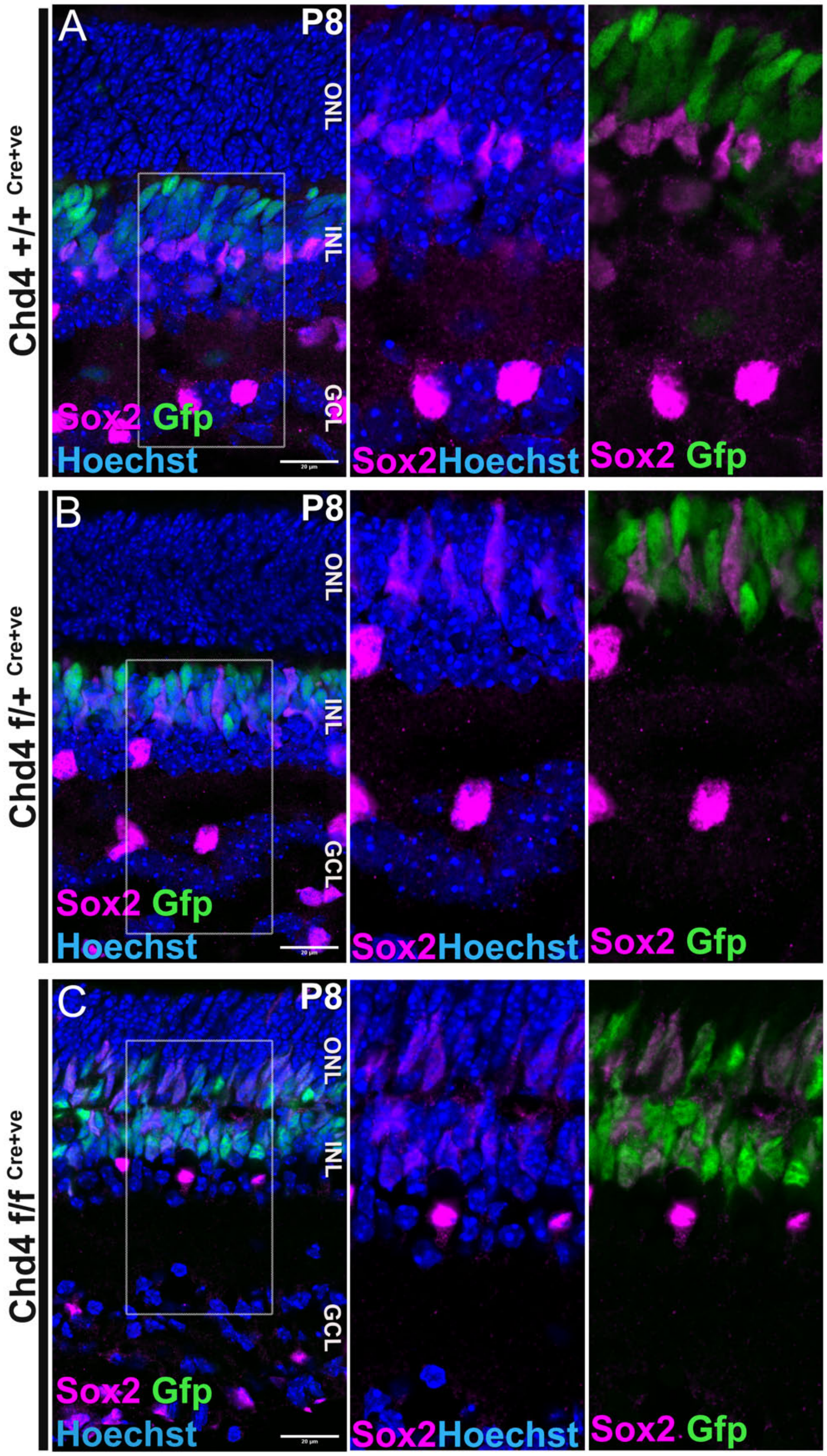

Fig. S9. Sox2 stainings at P8. In the basal INL and GCL, Sox2 marks early-born cholinergic amacrine cells with large circular nuclei. More apically, Sox2 marks radially polarized GFP+ Müllers. (A) Wild-type. (B) chet. (C) cKO. Data are from Fig. 5L-N. Scale bar = 20 microns.

Development • Supplementary information

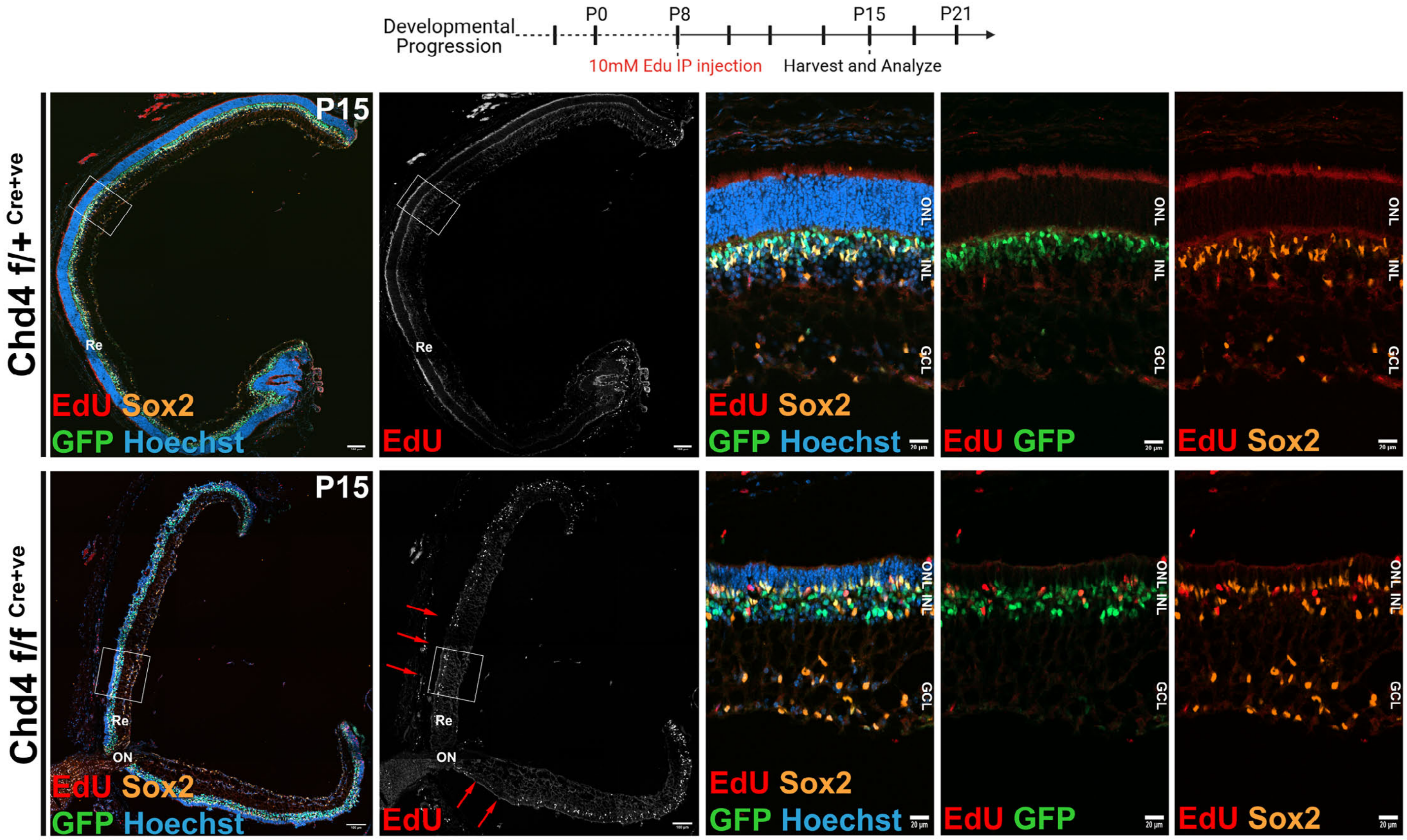

Fig. S10. Proliferating RPCs persist at late stages in the *Chd4* cKO, and mainly generate Sox2+ glia. EdU was injected at P8 and retinas were harvested at P15. Birthdated EdU+ cells were absent in the central retina in cHets (top). By contrast, EdU+ cells were observed throughout central retinal regions in cKO retinas (bottom). Boxed regions indicate the magnified areas shown in the rightmost panels.

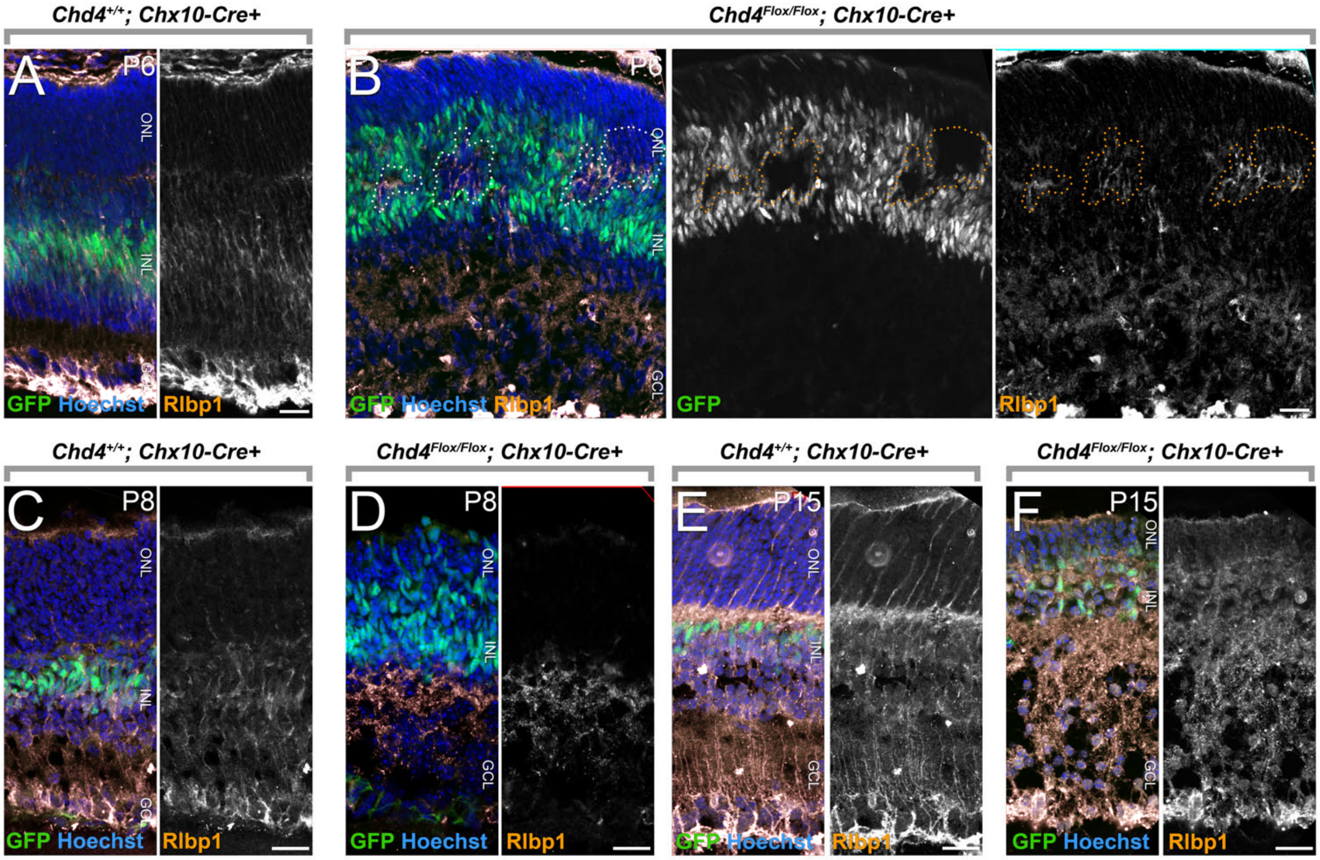

Fig. S11. Delayed glial differentiation in the *Chd4* cKO. (A-F) Rlbp1 staining on wild-type (A, C, E) versus cKO (B, D, F) retinas at P6 (A, B), P8 (C, D), and P15 (E, F). Retina in (B) includes patches of mosaicism (dotted outlines) as indicated by the lack of GFP expression. Scale bar = 20 microns.

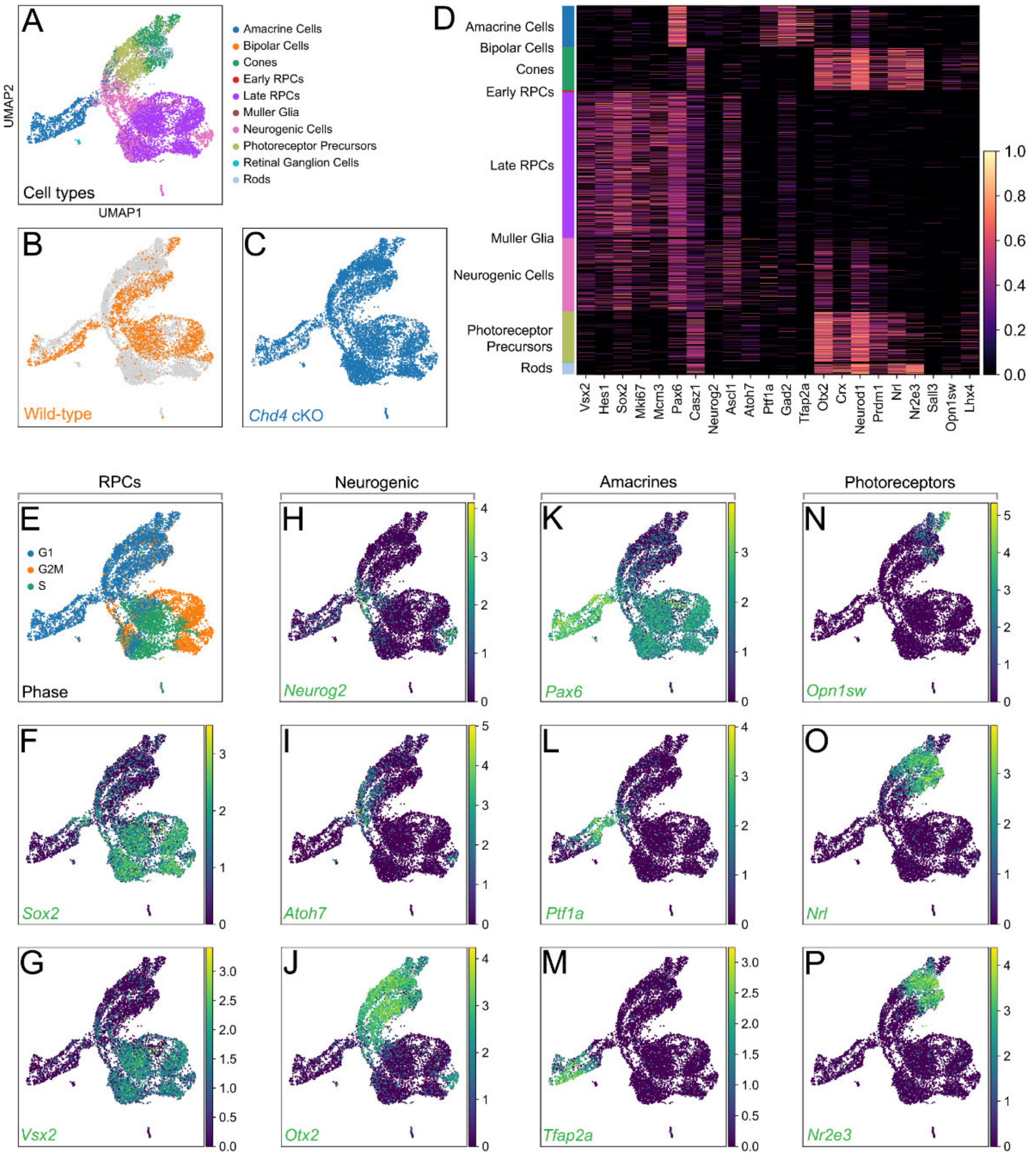

Fig. S12. Annotation of UMAP clusters using marker gene expression. (A) Cell-type annotation using label transfer from a published retinal scRNA-seq atlas (Clark et al., 2019). (B-C) Segregating the UMAP projection based on cell genotype. (D) Heat map of the expression of marker genes used to annotate the different cell type clusters present in the P1 scRNA-seq dataset. (E-P) UMAP projections of marker gene expression in control and *Chd4* cKO replicates. (E-G) RPC markers are based on the cell-cycle phase

Development • Supplementary information

(E), along with the expression of progenitor-specific markers *Sox2* (F) and *Vsx2* (G). (H-J) Markers of neurogenic cells determined by the expression of *Neurog2* (H), *Atoh7* (I), and *Otx2* (J). (K-M) Amacrine cell type markers *Pax6* (K), *Ptf1a* (L), and *Tfap2a* (M). (N-P) Photoreceptor markers *Opn1sw* (N), *Nrl* (O), and Nr2e3 (P).

Development • Supplementary information

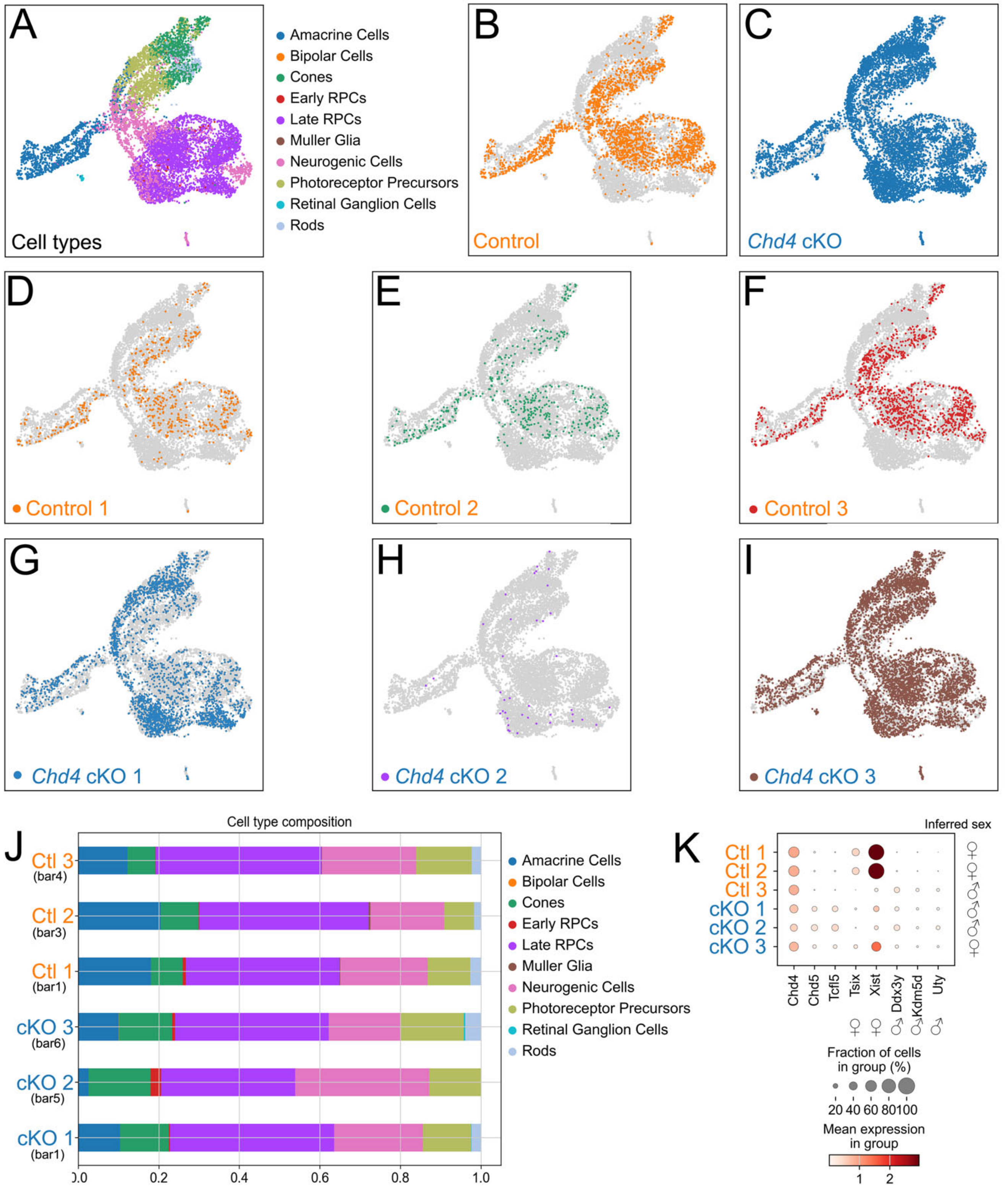

Fig. S13. Comparison of scRNA-seq replicates. (A) Cell-type annotation using label transfer from a published retinal scRNA-seq atlas (Clark et al., 2019). (B-C) UMAP projection of cells segregated by genotype. (D-I) UMAP projection of cells segregated by replicate. (J) Cell type proportions by replicate.

Development • Supplementary information

(K) Dotplot of gene expression by replicate. Note that *Chd4* cKO replicate 3 exhibits many cells that overlap with wild-type cells in UMAP space (I), suggesting that is likely mosaic. Accordingly, *Chd4* transcript is less reduced in this replicate, and the *Chd5* and *Tcfl5* transcripts that upregulate in the cKO are also less upregulated (K). Note also that the *Xist* and *Tsix* genes are transcribed from the inactivated X chromosome only in female cells, while *Ddx3y*, *Kdm5d*, and *Uty* genes are located only on the Y chromosome.

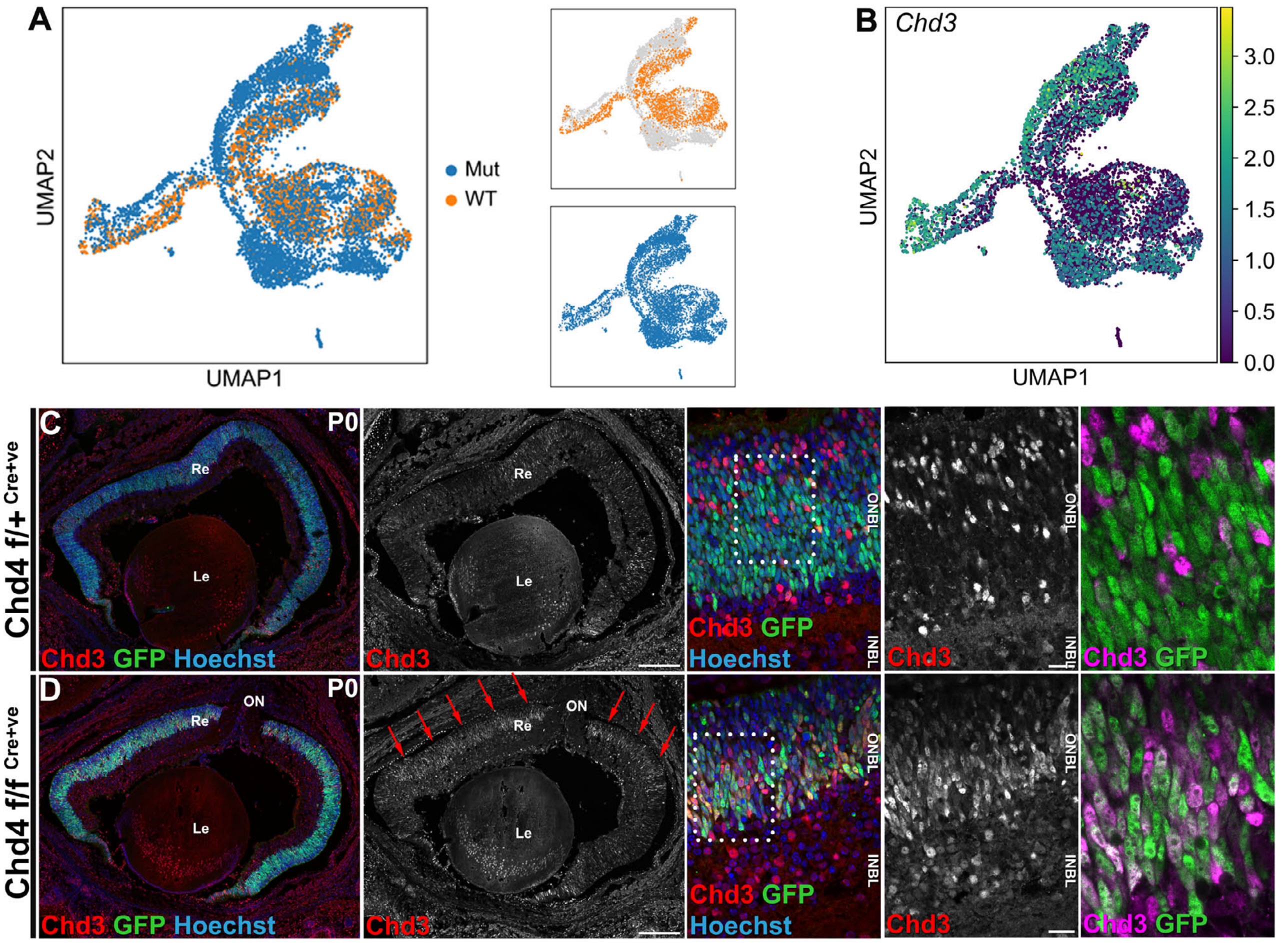

Fig. S14. Validation of the upregulation in Chd3 expression in the absence of *Chd4*. (A) UMAP representation of clusters segregated by genotype. (B) UMAP projection of *Chd3* expression in control and cKO samples. (C-D) IHC staining of Chd3 in chet and cKO P1 retinal sections.

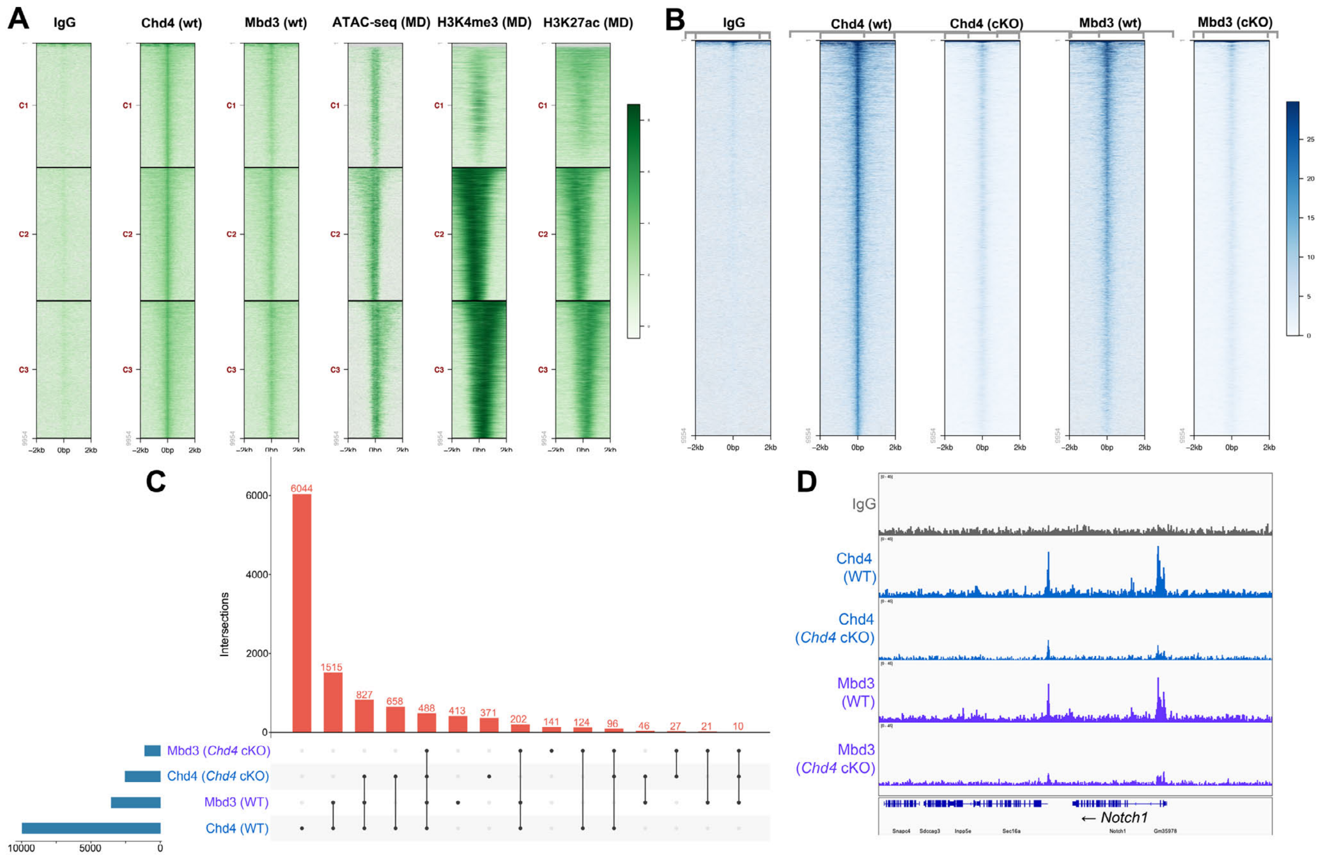

Fig. S15. Genomic occupancy of Chd4. (A) Heat map of Cut&Run-seq peaks of IgG, Chd4 and Mbd3 from wild-type and *Chd4* cKO P1 retinas centered on Chd4 wild-type peaks. (B) Comparing the wild-type cut&run-seq dataset with previously published ChIP-seq data (Aldiri et al., 2017) on age-matched retinas to determine the genomic occupancy of Chd4 and Mbd3. (C) Upset plot of cut&run-seq peak intersections. (D) IGV track of cut&run-seq peaks on the *Notch1* locus.

Development • Supplementary information

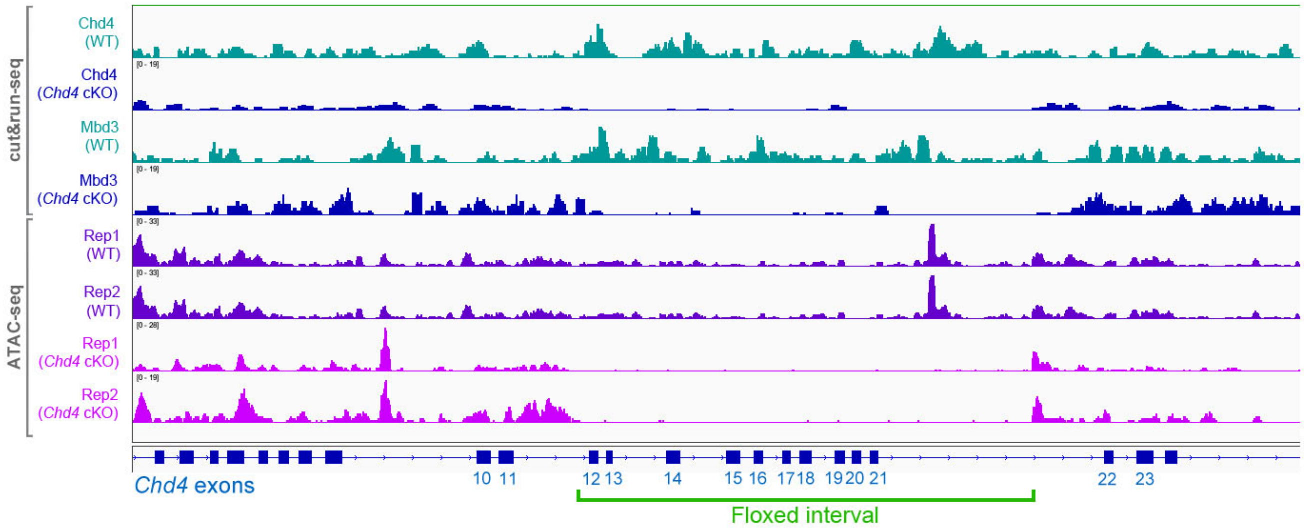

Fig. S16. Excision of the loxp flanked region in *Chd4* cKO genomic data.

Development • Supplementary information

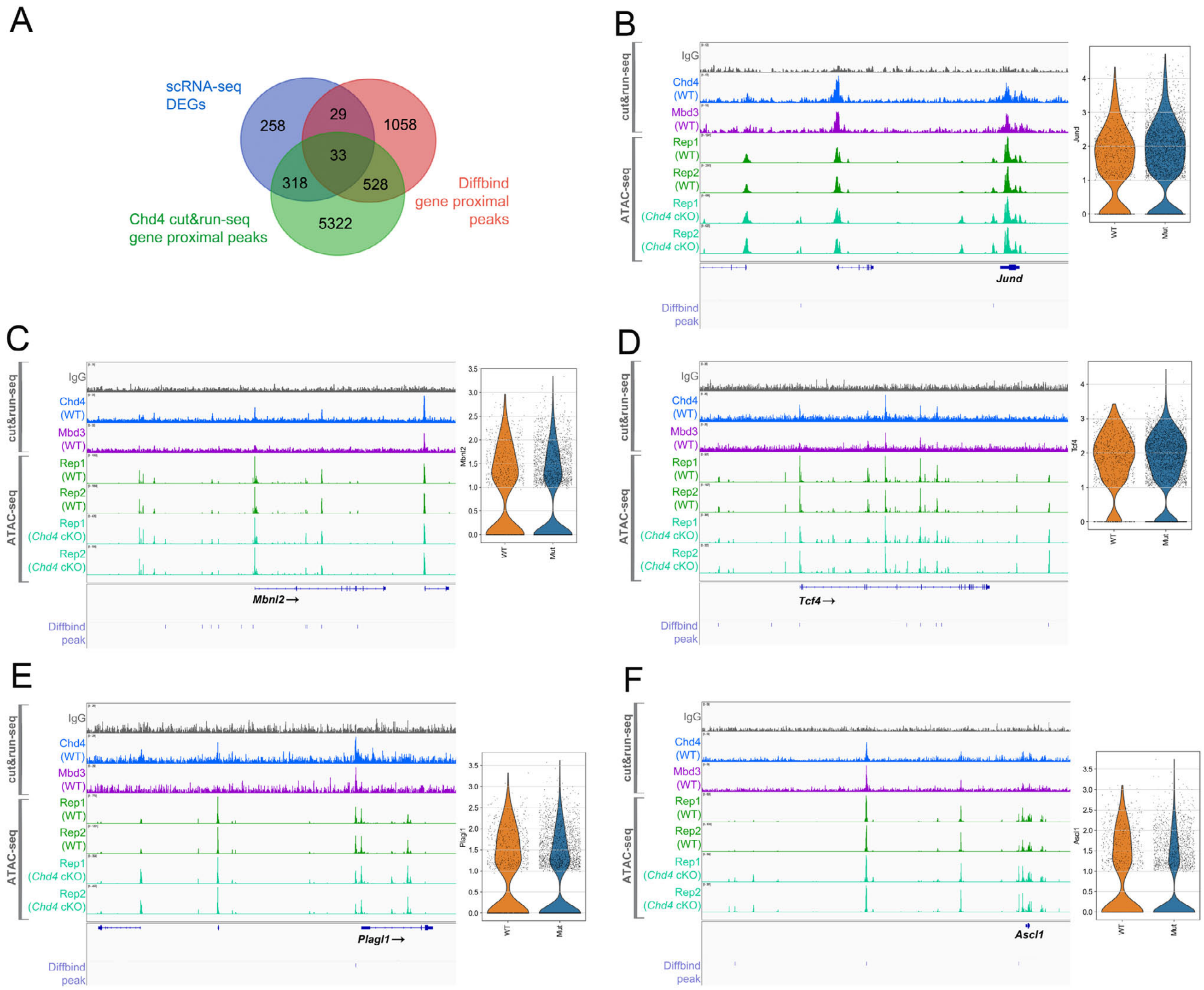

Fig. S17. (A) Integration of DEGs from the scRNA-seq analysis with genes associated with gene-proximal DARs and Chd4 Cut&Run-seq gene-proximal peaks. (B) Cut&Run-seq and ATAC-seq tracks for selected DEGs that are directly occupied by Chd4. Diffbind peaks indicate DARs from control vs. *Chd4* cKO ATAC-seq data. Violin plots display scRNA-seq expression data from late RPCs.

Development • Supplementary information

Table S1. Oligonucleotide sequences. Multi-seq barcodes are highlighted in green.

Name	Purpose	Sequence
Mi-2β+F S	genotyping	5′-CTCCAAGAAGAAGACGGCAGATCT-3′
Mi-2 INR A	genotyping	5′-GTCCTTCCAAGAAGAGCAAG-3′
CRE-F	genotyping	5′-AGGTGTAGAGAAGGCACTTAGC-3′
CRE-R	genotyping	5′-CTAATCGCCATCTTCCAGCAGG-3′
Multiseq-1	barcoding	5′-CCTTGGCACCCGAGAATTCCAGGAGAAGAAAAAAAAAAAAAAAAAAAAAAAAAAAAAAA-3′
Multiseq-2	barcoding	5′-CCTTGGCACCCGAGAATTCCACCACAATGAAAAAAAAAAAAAAAAAAAAAAAAAAAAAA-3′
Multiseq-3	barcoding	5′-CCTTGGCACCCGAGAATTCCATGAGACCTAAAAAAAAAAAAAAAAAAAAAAAAAAAAAA-3′
Multiseq-4	barcoding	5′-CCTTGGCACCCGAGAATTCCAGCACACGCAAAAAAAAAAAAAAAAAAAAAAAAAAAAAA-3′
Multiseq-5	barcoding	5′-CCTTGGCACCCGAGAATTCCAAGAGAGAGAAAAAAAAAAAAAAAAAAAAAAAAAAAAAA-3′
Multiseq-6	barcoding	5′-CCTTGGCACCCGAGAATTCCATCACAGCAAAAAAAAAAAAAAAAAAAAAAAAAAAAAAA-3′

Development • Supplementary information

Table S2. Antibodies and dilutions.

Antigen	Species	Dilution	Supplier	Catalog	RRID	Application
Brn3a	Mouse	1:100	Millipore Sigma	MAB1585MI	AB_94166	IHC
Brn3b	Goat	1:200	Santa Cruz	sc-6026	AB_673441	IHC
Chd3	Rabbit	1:200	Fortis	A301-220A-T	AB_890570	IHC
Chd4	Rat	1:200	Biolegend	942302	AB_2888898	IHC
Chd4	Rabbit	1:1000<br>1:100	Abcam	ab72418	AB_1268107	Western,<br>cut&run
Cleaved Caspase-3 (Asp175)	Rabbit	1:500	Cell Signaling	9579	AB_10897512	IHC
Cone arrestin	Rabbit	1:200	Millipore Sigma	AB15282	AB_1163387	IHC
GFP	Mouse	1:100	DSHB	GFP-G1	AB_2619561	IHC
Ki67	Rabbit	1:100	Millipore Sigma	SAB5500134	AB_2892217	IHC
Lhx2	Rabbit	1:200	Millipore Sigma	ABE1402	AB_2722523	IHC
Mbd3	Rabbit	1:100	Abcam	EPR18258	Not found	cut&run
Neurofilament	Mouse	1:100	DSHB	2H3	AB_531793	IHC
Nr2e3	Mouse	1:100	R&D Systems	PP-H7223-00	AB_2155481	IHC
Otx2	Goat	1:500	R&D Systems	AF1979	AB_2157172	IHC
Pax6	Rabbit	1:500	Novus	NBP2-19711	AB_3264565	IHC
Pax6	Rabbit	1:500	Proteintech	12323-1-AP	AB_2159695	IHC
pHH3	Rabbit	1:500	Cell Signaling	9701S	AB_331535	IHC
Rbpms	Guinea pig	1:200	Millipore Sigma	ABN1376	AB_2687403	IHC
Rlbp1	Mouse	1:100	Invitrogen	MA1-813	AB_2178528	IHC
Rxrγ	Mouse	1:100	Santa Cruz	sc-365252	AB_10850062	IHC
Sox2	Goat	1:500	R&D Systems	AF2018-SP	AB_355110	IHC
Tfap2a	Mouse	1:500	DSHB	3B5	AB_2313948	IHC
anti-guinea pig Alexa Fluor™ 555	Goat	1:1000	Rockland	606-142-129	AB_1961616	2º (IHC)
anti-goat Alexa Fluor™ 488	Donkey	1:1000	Jackson	705-547-003	AB_2340431	2º (IHC)
anti-goat DyLight™ 650	Donkey	1:1000	Novus	NBP1-75604	AB_11018252	2º (IHC)
anti-mouse Alexa Fluor™ 555	Donkey	1:1000	Invitrogen	A-31570	AB_2536180	2º (IHC)
anti-mouse Alexa Fluor™ 647	Donkey	1:1000	Invitrogen	A-31571	AB_162542	2º (IHC)
anti-rabbit Alexa Fluor™ 488	Donkey	1:1000	Jackson	111-545-003	AB_2338046	2º (IHC)
anti-rabbit Alexa Fluor™ 647	Donkey	1:1000	Jackson	711-607-003	AB_2340626	2º (IHC)
anti-rat DyLight™ 550	Donkey	1:1000	Invitrogen	SA510027	AB_2556607	2º (IHC)
anti-Rabbit HRP	Donkey	1:10 000	GE Healthcare	NA934	AB_772206	2º<br>(Western)

Development • Supplementary information

Table S3. Genomic and Transcriptomic datasets. Tab 1: Differential gene expression in 'Late RPCs'. Tab 2: Differential gene expression in all cells (pseudobulk). Tab 3: List of gene-proximal DARs as determined using the GREAT algorithm (McLean et al., 2010). Tab 4: Differential gene body accessibility as determined via LIMMA (Law et al., 2014). Tab 5: Gene list intersection for DEGs, gene proximal DARs, and cut&run peaks.

Available for download at
https://journals.biologists.com/dev/article-lookup/doi/10.1242/dev.204697#supplementary-data

References

Aldiri, I., Xu, B., Wang, L., Chen, X., Hiler, D., Griffiths, L., Valentine, M., Shirinifard, A., Thiagarajan, S., Sablauer, A., et al. (2017). The Dynamic Epigenetic Landscape of the Retina During Development, Reprogramming, and Tumorigenesis. *Neuron* **94**, 550-568 e510.

Clark, B. S., Stein-O'Brien, G. L., Shiau, F., Cannon, G. H., Davis-Marcisak, E., Sherman, T., Santiago, C. P., Hoang, T. V., Rajaii, F., James-Esposito, R. E., et al. (2019). Single-Cell RNA-Seq Analysis of Retinal Development Identifies NFI Factors as Regulating Mitotic Exit and Late-Born Cell Specification. *Neuron* **102**, 1111-1126 e1115.

Law, C. W., Chen, Y., Shi, W. and Smyth, G. K. (2014). voom: Precision weights unlock linear model analysis tools for RNA-seq read counts. *Genome Biol* **15**, R29.

McLean, C. Y., Bristor, D., Hiller, M., Clarke, S. L., Schaar, B. T., Lowe, C. B., Wenger, A. M. and Bejerano, G. (2010). GREAT improves functional interpretation of cis-regulatory regions. *Nat Biotechnol* **28**, 495-501.
